# Intensity modulated radiotherapy for elderly bladder cancer patients

**DOI:** 10.1186/1748-717X-6-75

**Published:** 2011-06-16

**Authors:** Chen-Hsi Hsieh, Shiu-Dong Chung, Pei-Hui Chan, Siu-Kai Lai, Hsiao-Chun Chang, Chi-Huang Hsiao, Le-Jung Wu, Ngot-Swan Chong, Yu-Jen Chen, Li-Ying Wang, Yen-Ping Hsieh, Pei-Wei Shueng

**Affiliations:** 1 Division of Radiation Oncology, Department of Radiology, Far Eastern Memorial Hospital, Taipei, Taiwan; 2 Division of Urology, Far Eastern Memorial Hospital, Taipei, Taiwan; 3 Division of Medical Oncology and Hematology, Department of Internal Medicine, Far Eastern Memorial Hospital, Taipei, Taiwan; 4 Department of Radiation Oncology, Mackay Memorial Hospital, Taipei, Taiwan; 5 Department of Medical Research, Mackay Memorial Hospital, Taipei, Taiwan; 6 Department of Radiation Oncology, National Defense Medical Center, Taipei, Taiwan; 7 Institute of Traditional Medicine, School of Medicine, National Yang-Ming University, Taipei, Taiwan; 8 Graduate Institute of Sport Coaching Science, Chinese Culture University, Taipei, Taiwan; 9 School and Graduate Institute of Physical Therapy, College of Medicine, National Taiwan University, Taipei, Taiwan; 10 Department of Healthcare Administration, Asia University, Taichung, Taiwan

**Keywords:** Bladder cancer, Concurrent chemoradiation, Helical tomotherapy, Intensity modulated radiation therapy

## Abstract

**Background:**

To review our experience and evaluate treatment planning using intensity-modulated radiotherapy (IMRT) and helical tomotherapy (HT) for the treatment of elderly patients with bladder cancer.

**Methods:**

From November 2006 through November 2009, we enrolled 19 elderly patients with histologically confirmed bladder cancer, 9 in the IMRT and 10 in the HT group. The patients received 64.8 Gy to the bladder with or without concurrent chemotherapy. Conventional 4-field "box" pelvic radiation therapy (2DRT) plans were generated for comparison.

**Results:**

The median patient age was 80 years old (range, 65-90 years old). The median survival was 21 months (5 to 26 months). The actuarial 2-year overall survival (OS) for the IMRT vs. the HT group was 26.3% *vs *.37.5%, respectively; the corresponding values for disease-free survival were 58.3% *vs*. 83.3%, respectively; for locoregional progression-free survival (LRPFS), the values were 87.5% *vs*. 83.3%, respectively; and for metastases-free survival, the values were 66.7% *vs*. 60.0%, respectively. The 2-year OS rates for T1, 2 *vs*. T3, 4 were 66.7% *vs*. 35.4%, respectively (*p *= 0.046). The 2-year OS rate was poor for those whose RT completion time greater than 8 weeks when compared with the RT completed within 8 wks (37.9% vs. 0%, *p *= 0.004).

**Conclusion:**

IMRT and HT provide good LRPFS with tolerable toxicity for elderly patients with invasive bladder cancer. IMRT and HT dosimetry and organ sparing capability were superior to that of 2DRT, and HT provides better sparing ability than IMRT. The T category and the RT completion time influence OS rate.

## Background

Radical cystectomy with pelvic lymph node dissection has long been the standard of care for invasive bladder cancer. However, the procedure involves removal of the bladder, surrounding structures (including the prostate gland or uterus), regional lymph nodes, with urinary diversion. Accordingly, radical cystectomy often results in considerable morbidity, including incontinence and impotence [1,2]. Due to the potential morbidity and for patients whose conditions are not amenable to curative treatment and for whom palliative treatment (organ preservation) is the best choice, multiple modalities have been the topic of recent investigations. There are several groups that have reported the value of combined-modality therapy, including transurethral resection (TURBT) of the bladder tumor, radiation therapy (RT), and systemic chemotherapy [3-7]. The elderly patients, however, may have age-related changes in their physiology, which alter their tolerance to full course radiotherapy and are generally medically unfit for cystectomy [8].

The morbidity in the bladder cancer treated with RT is well known [9]. Of the patients treated with RT, 45.7% had severe reactions in the bladder and 8.5% had severe reactions in the bowel. Of the Radiation Therapy Oncology Group (RTOG) patients, 7% experienced late grade 3+ pelvic toxicity [10]. In the initial results of RTOG 95-06, 21% of patients with muscle-invading bladder cancer developed grade 3 or 4 hematologic toxicity with TURBT plus concurrent chemoradiation therapy (CCRT) [11]. The RT technique used in these reports was conventional RT, in which the dose cannot be reduced to critical organs, and thus, causes unavoidable side effects.

For tumors located in the pelvis improvements in treatment planning and delivery have evolved from conventional to intensity-modulated radiotherapy (IMRT) [12]. For example, under similar target coverage, IMRT is superior to conventional techniques in normal tissue sparing for the treatment of cervical cancer, and a number of groups have explored IMRT in the gynecologic setting as a method to minimize the gastrointestinal, genitourinary, and bone marrow toxicity that occurs with conventional RT [13-15].

Helical tomotherapy (HT), an image-guided IMRT, delivers highly conformal dose distributions to the targets, with simultaneous critical organ sparing [15,16]. Owing to the shape and location, the extent of bladder tumors make them well suited for HT. In our institute, a Tomotherapy Hi-Art System (Tomotherapy, Inc., Madison, Wisconsin, USA) was placed into service in November 2006. We report our initial clinical experience with bladder cancer patients treated with IMRT or HT for organ preservation, focusing on feasibility of IMRT and HT, clinical outcome, and early toxicities.

## Methods

### Patient characteristics

Between November 2006 and November 2009, we retrospectively reviewed the medical records of 25 patients with muscle-invasive (T2 to T4) or high-risk T1-bladder cancer were treated with either RT (n = 12) alone or with CCRT (n = 13) after initial TURBT of the tumor. Risk factors for T1-cancer were defined as tumor grade 3/4, associated carcinoma-in situ, multifocal tumors, or recurrent tumors refractory to repeated TURBT with or without intravesical therapy. Excluded from analysis were six patients in whom treatment was regarded to be palliative because of concomitant distant disease or in which the radiation dose to the bladder was insufficient (less than 45 Gy), or they were younger than 65 years. All of the remaining 19 patients (9 who had IMRT and 10 who had HT) were free of distant metastases at the time of RT/CCRT. Pelvic lymph node metastases (detected by computed tomography or ultrasound), multiple TURBTs before RT/CCRT, or poor general condition with contraindications for radical cystectomy were not considered exclusion criteria. Patient and tumor characteristics are listed in Table 1. The disease was staged according to the American Joint Committee on Cancer staging classifications 6^th ^edition.

**Table 1:** Patient characteristics

	IMRT	HT	All
	
	No. of patient (%)		
**Age **(years)			
Mean	79.9	78.5	79.2
Range	66-90	65-89	65-90
**Gender**			
Male	7 (77.8%)	7 (70%)	14 (73.7%)
Female	2 (22.2%)	3 (30%)	5 (26.3%)
**Karnofsky performance status**			
< 60	0	0	0
≥ 60	9 (100%)	10 (100%)	19 (100%)
**Pathology**			
Urothelial carcinoma	9 (100%)	10 (100%)	19 (100%)
**Tumor stage**			
Stage I	0	1 (10%)	1 (5.3%)
Stage II	2 (22.2%)	2 (20%)	4 (21.1%)
Stage III	4 (44.4%)	4 (40%)	8 (42.1%)
Stage IV	3 (33.3%)	3 (30%)	6 (31.6%)
**Primary Tumor stage**			
T1-high risk	0	1 (10%)	1 (5.3%)
T2	2 (22.2%)	3 (30%)	5 (26.3%)
T3	5 (55.6%)	3 (30%)	8 (42.1%)
T4	2 (22.2%)	3 (30%)	5 (26.3%)
**Regional Lymph Node stage**			
N0	6 (66.7%)	7 (70%)	13 (68.4%)
N1	2 (22.2%)	1 (10%)	3 (15.8%)
N2	1 (11.1%)	2 (20%)	3 (15.8%)
**Concurrent with chemotherapy**	3 (33.3%)	6 (60%)	9 (47.4%)
**Median dose for RT completion (range) (Gy)**	57.6 (45-64.8)	57.6 (54-64.8)	57.6 (45-64.8)
**Median time for RT completion (range) (wks)**	7 (6-11)	6.5 (5-10)	7 (5-11)

Abbreviations:

All = all Patients in the study; HT = helical tomotherapy; IMRT = intensity-modulated radiation therapy; RT = radiation therapy

### Radiotherapy

RT/CCRT was initiated 4 to 8 weeks after initial TURBTs using 6-MV photons and a 7-filed IMRT or HT technique with daily fractions of 1.8 Gy in five consecutive days. A total of 10 patients were treated by RT alone (six with IMRT and four with HT). Chemotherapy was given during RT and consisted of weekly cisplatin (30 mg/m^2^) in three patients or carboplatin (area under the curve [AUC] of 4-6 mg/mL. min) every 21 days in two patients with decreased creatinine clearance (< 60 mL/min) or congestive heart disease. A combination of weekly cisplatin (30 mg/m^2^) and weekly 5-fluorouracil (5-FU) (450 mg/m^2^) was given to one patient. A combination of gemcitabine (800-1000 mg/m^2^) and carboplatin (AUC of 4-6 mg/mL. min) on days 1 and 8 of a 3-week cycle was given to three patients (Table 1).

### Immobilization

The BlueBAG™ immobilization system (Medical Intelligence, Schwabmünchen, Germany) was used to immobilize the pelvis and extremities. Positioning was supine with arms folded across the chest with ankle supports. The bladder was emptied immediately before scanning and treatment. All patients underwent a 5-mm slice thickness CT planning scan (Siemens Somatom Plus 4 CT scanner) from the L1 to 5 cm below the ischial tuberosities. Target objects and normal structures were contoured with a Pinnacle 3 treatment planning system (Philips Healthcare, Madison, Wisconsin, USA). The MRI or CT images were retrieved on a Pinnacle workstation and fused with the CT images for contouring of the tumor volume.

### Delineation of target volumes

The gross tumor volume (GTV) was defined as all known gross disease determined by CT, clinical information, and MRI. The clinical target volume (CTV) was defined as the GTV, the whole bladder, and pelvic lymph nodes [17-19]. In patients with tumors at the bladder base, the proximal urethra, and in men, the prostate and prostatic urethra, were included in the CTV. The nodal CTV included the internal (hypogastric and obturator) and external iliac lymph nodes and perinodal tissue [20]. Seven mm was extended from the vessels as the margin of nodal CTV. Bone and intraperitoneal small bowel was excluded from the nodal CTV; in addition, the iliopsoas muscle that lies adjacent to clinically negative lymph nodes was also excluded from the nodal CTV. The most antero-lateral external iliac lymph nodes positioned just proximal to the inguinal canal were excluded from the nodal CTV. The CTV of the nodes ended 7 mm from the L5/S1 interspace to account for the PTV. The PTV for nodes stopped at the L5/S1 interspace. The planning target volume (PTV) provided a 7-mm margin (anteriorly, posteriorly, laterally, as well as superiorly and inferiorly) around the nodal CTV as PTV _nodal _[15] and a 1 to 1.5-cm margin for CTV as PTV [21-23]. The sequential boost field of CTV was defined as the GTV (primary tumor and any extravesical spread). The boost field of the PTV consisted of a 1.5-cm margin around the CTV boost edges except superiorly where the extension was 2.5 cm. These margins incorporated internal margins and set-up margins [24,25]. The treatment plan used for each patient was based on an analysis of the volumetric dose, including dose volume histogram (DVH) analyses of the PTV and critical normal structures.

The 90% isodose surface covered between 95% and 98% of the PTV, or volumes of overdose exceeding 115% < 5% of the PTV volume were considered acceptable. The field width, pitch, and modulation factor usually used for the HT treatment planning optimization were 2.5 cm, 0.32, and 3.0, respectively. All HT-treated patients received daily megavoltage computed tomography (MVCT) acquisitions for setup verification [26]. The organs at risk (OARs) were contoured using the empty-bladder CT scan. Dose-volume constraints for normal tissues were as follows: small bowel (2 cm above the most superior vessel contour) 250 cc received < 45 Gy; femoral head V30 < 15%; rectum V30 < 50%, V55 < 10%. The rectum volume was defined on CT from the anus (at the level of the ischial tuberosities) for a length of 15 cm, or to the rectosigmoid flexure.

### Conventional treatment planning for comparison

Conventional whole pelvic radiation therapy (2DRT) plans were generated using the Pinnacle 3 Treatment Planning System (Philips Healthcare, Madison, Wisconsin, USA). A 4-field "box" plan was designed using 6-MV photons with apertures shaped to the PTV in each beam's eye-view. The field margins in the inferior and superior dimensions extended 1 cm below the lower pole of the obturator foramen to the mid-sacrum (the anterior aspect of the S1-S2 junction). Laterally, the anterior and posterior opposed fields extended at least 1.5 cm beyond the widest point of the bony margin of the pelvis. For the parallel opposed lateral fields, the field edges extended 3.0 cm posterior to the CTV bladder and extended 1 cm anterior to the most anterior point of the symphysis pubis or 1.5 cm anterior to the anterior tip of the bladder, whichever was the most anterior. Superiorly, the lateral fields included blocks anteriorly to exclude the small bowel and the anterior rectus fascia. At least 98% of the PTV were encompassed by the prescribe doses.

### Dose-volume analysis of treatment plans

The conformity index (CI) was originally proposed by Paddick [27] to evaluate the tightness of fit of the planning target volume to the prescription isodose volume in treatment plans as follows,(1)

where V*_PTV _*is the volume of the PTV, V*_TV _*is the treated volume enclosed by the prescription isodose surface, and *TV_PV _*is the portion of the PTV within the prescribed isodose volume. The uniformity index (UI) was defined as *D_5%_/D_95%_*, where D_5% _and D_95% _were the minimum doses delivered to 5% and 95% of the PTV, as previously reported [28].

### Toxicity

Interruptions in radiotherapy could be necessitated by uncontrolled diarrhea, or other acute complications. If radiation therapy was temporarily stopped, then chemotherapy was also stopped. Chemotherapy was normally stopped at the completion of RT. If chemotherapy was stopped, RT would continue. RT was only stopped in cases of grade 4 hematologic or non-hematologic toxicity until toxicity resolved at least to grade 3 or less. Chemotherapy was withheld in any case involving grade 3 toxicity until the toxicity regressed to any grade of < 3; in patients with grade 3 toxicity that persisted longer than 2 weeks, chemotherapy was no longer administered.

### Follow-up

Upon treatment completion, patients were evaluated every 3 months for the first year, every 4 months during the second year, every 6 months during the third year, and annually thereafter. At each visit, a physical and pelvic examination, blood counts, clinical chemistry, chest x-rays and cystoscopies were performed. CT, ultrasonography, and other imaging studies were conducted when appropriate. Suspected cases of persistent or recurrent disease were confirmed by biopsy whenever possible. Acute and late toxicities (occurring > 90 days after beginning RT) were defined and graded according to the Common Terminology Criteria for Adverse Events v3.0.

### Statistical methods

Descriptive statistics (means, medians, and proportions) were calculated to characterize the patient, disease, and treatment features as well as toxicities after treatment. The overall survival (OS), progression-free survival (PFS), locoregional progression-free survival (LRPFS), and metastases-free survival (MFS) rates were estimated using the Kaplan-Meier product-limit method. Progression was defined as a 50% increase in the product of the two largest diameters of the primary tumor or metastasis. Progression-free survival was calculated from the date of pathologic proof to the date of the first physical or radiographic evidence of disease progression, death, or the last follow-up visit. Survival was calculated from the date of pathologic proof to the date of death or the last follow-up visit. All analyses were performed using SPSS, version 12.0 (SPSS, Chicago, IL, USA).

## Results

### Patient characteristics

Table 1 details the patient characteristics. Fourteen men and five women were included (nine in the IMRT group and 10 in the HT group). They had a median age of 80 years (range, 65-90 years). All patients had urothelial carcinoma. Only 5% of the patients had a T1 high-risk primary tumor, while 95% had T2-4 tumors; 32% were node positive. The disease stage distribution was as follows: 1 Stage I (5%), 4 Stage II (21%), 8 Stage III (42%), and 6 Stage IV (32%). The median dose of RT for all, IMRT- and HT-treated group was 57.6 Gy. The median duration of RT for all, IMRT- and HT-treated groups was 7, 7 and 6.5 weeks, respectively. The characteristics of patients in the IMRT and HT groups were similar (Table 1).

### Treatment outcome

**Figure 1:**
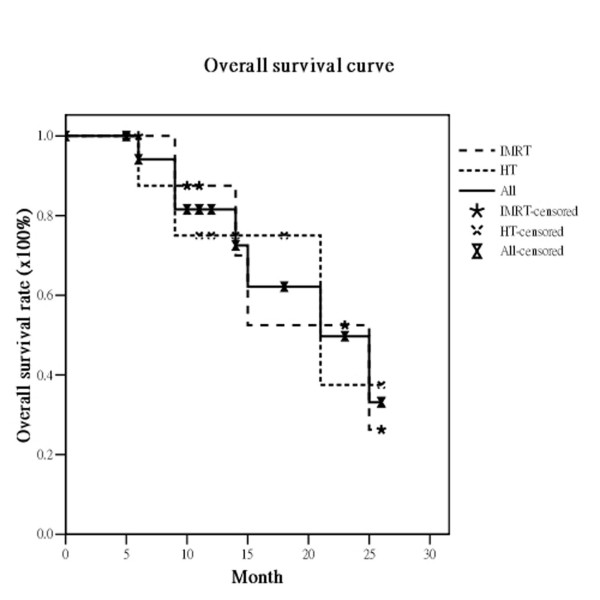
**The actuarial overall survival rates at 2 years for all bladder cancer patients and the patients treated with intensity-modulated radiation therapy (IMRT) and helical tomotherapy (HT)**.

**Figure 2:**
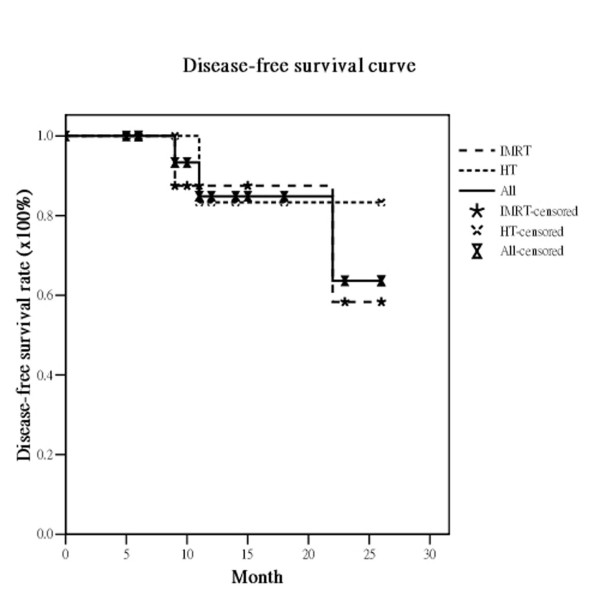
**The actuarial disease-free survival rates at 2 years for all bladder cancer patients and the patients treated with intensity-modulated radiation therapy (IMRT) and helical tomotherapy (HT)**.

**Figure 3:**
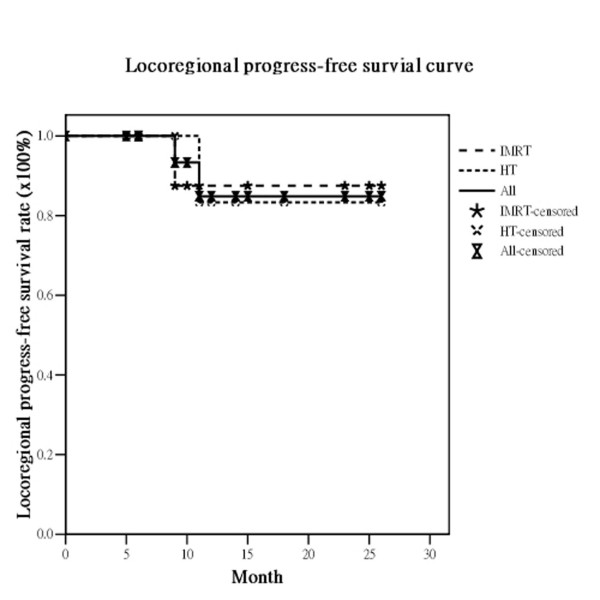
**The actuarial locoregional progress-free survival rates at 2 years for all bladder cancer patients and the patients treated with intensity-modulated radiation therapy (IMRT) and helical tomotherapy (HT)**.

**Figure 4:**
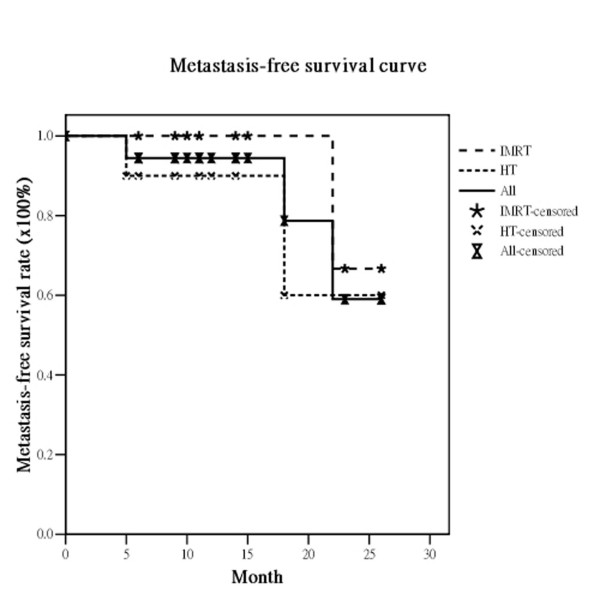
**The actuarial metastasis-free survival rates at 2 years for all bladder cancer patients and the patients treated with intensity-modulated radiation therapy (IMRT) and helical tomotherapy (HT)**.

The median survival was 21 months (range, 5-26 months). Of the 19 eligible patients, 17 (89.5%) had no local recurrence. Only two patients experienced recurrence, one in the IMRT and one in the HT group. The actuarial 2-year OS, DFS, LRPFS, and MFS for all *vs*. the IMRT group *vs*. the HT group were 33.2% *vs*. 26.3% *vs*. 37.5%, 63.6% *vs*. 58.3% *vs*. 83.3%, 84.9% *vs*. 87.5% *vs*. 83.3% and 59.0% *vs*. 66.7% *vs*. 60.0%, respectively (Figure 1, Figure 2, Figure 3, Figure 4). There are not statistically differences between both groups about OS, DFS, LRPFS, and MFS. T stage affected the OS rate of the elderly, which for T1/2 *vs*. T3/4 was 66.7% *vs*. 35.4% (*p *= 0.046). The 2-year OS rate for stage I/II *vs*. stage III/IV was 66.7% *vs*. 39.1% (*p *= 0.07) There were 4 patients with RT completion times greater than 8 wks (In IMRT group: one is 10 wks and the other is 11 wks; In HT group: one is 9 wks and the other is 10 wks) and The patients with RT completion times greater than 8 weeks had poorer 2-year OS rates (37.9% vs. 0%, *p *= 0.004).

### Dose-volume analysis

**Figure 5:**
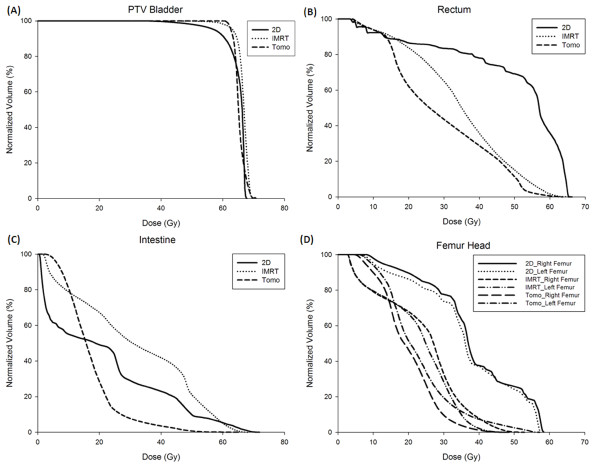
**The comparisons of dose-volume histogram of planning target volume (PTV) and organs at risk for one of intensity-modulated radiation therapy (IMRT) - treated patients, one of helical tomotherapy (HT) - treated patients and one of the patients replanned by conventional box techniques (2DRT)**. (A) PTV. (B) Rectum. (C) Intestine. (D) Femur head.

Comparing 2DRT with IMRT and HT, the UI and CI were 1.10 ± 0.03 vs. 1.09 ± 0.01 vs. 1.01 ± 0.01 and 3.17 ± 1.01 vs. 1.22 ± 0.06 vs. 1.20 ± 0.03, respectively. The mean of V30 for the right and left side femoral heads for the three RT modalities were 74% vs. 35% vs. 6% and 71% vs.26.5% vs. 6%, respectively. The mean radiation dosages (Gy) to the rectum and intestines for the three RT modalities were 50 Gy vs. 34 Gy vs. 25 Gy and 40 Gy vs. 29 Gy vs. 21 Gy, respectively. The comparisons of dose-volume histogram statistics for the organs at risk (OARs) are described in Table 2 and Figure 5.

**Table 2:** Comparison of dosimetric parameters for irradiation of bladder cancer and normal organs at risk (OARs) by using different treatment techniques.

		IMRT	HT	2DRT	P value
**PTV**	UI	1.09 ± 0.01	1.01 ± 0.01	1.10 ± 0.03	IMRT vs. HT: *p *= 0.001 2DRT vs. IMRT: *p *= 0.19 2DRT vs. HT: *p *= 0.002
	CI	1.22 ± 0.06	1.20 ± 0.03	3.17 ± 1.01	IMRT vs. HT: *p *= 0.19 2DRT vs. IMRT: *p *< 0.001 2DRT vs. HT: *p *< 0.001
**Right Femoral head (V30)**	mean (%)	35.0 ± 0.2	6.0 ± 0.1	73.7 ± 19.7	IMRT vs. HT: *p *= 0.001 2DRT vs. IMRT: *p *< 0.001 2DRT vs. HT: *p *< 0.001
**Left Femoral head (V30)**	mean (%)	26.5 ± 0.3	6.1 ± 0.1	71.1 ± 22.9	IMRT vs. HT: *p *= 0.03 2DRT vs. IMRT: *p *< 0.001 2DRT vs. HT: *p *< 0.001
**Rectum**	mean (Gy)	34.3 ± 9.1	25.4 ± 5.9	50.4 ± 8.1	IMRT vs. HT: *p *= 0.019 2DRT vs. IMRT: *p *< 0.001 2DRT vs. HT: *p *< 0.001
	V55_Gy _< 50% (%)	4.7 ± 9.6	1.4 ± 2.8	46.1 ± 36.8	IMRT vs. HT: *p *= 0.28 2DRT vs. IMRT: *p *= 0.001 2DRT vs. HT: *p *< 0.001
**Intestine**	mean (Gy)	29.2 ± 9.3	20.7 ± 6.6	40.2 ± 13.2	IMRT vs. HT: *p *= 0.034 2DRT vs. IMRT: *p *= 0.034 2DRT vs. HT: *p *< 0.001
	250 c.c. < 45 Gy (c.c.)	25.9 ± 30.1	10.8 ± 11.9	192.6 ± 132.6	IMRT vs. HT: *p *= 0.16 2DRT vs. IMRT: *p *= 0.001 2DRT vs. HT: *p *< 0.001

The Vx is the percentage of femoral head volume that receives ≥ X Gy in the total femoral head volume.

Abbreviations:

2DRT: Conventional whole pelvic radiation therapy; CI: Conformal index; IMRT = intensity-modulated radiation therapy; HT = helical tomotherapy; UI: Uniformity index.

### Acute toxicity

No grade 3 of acute toxicity for thrombocytopenia, diarrhea, and nausea/vomiting occurred in either group. Only one IMRT-treated patient suffered from grade 2 of diarrhea during treatment. The other IMRT-treated and HT-treated patients experienced grade 1 diarrhea and nausea/vomiting. In the IMRT-treated group, two patients experienced grade 3/4 anemia and two experienced grade 3 leukopenia. In the HT-treated group, only one patient experienced grade 3 anemia and no patient experienced grade 3 leukopenia.

## Discussion

This preliminary study showed that IMRT and HT both produce minimal grade 3 or greater toxicity and provide good LRPFS. This supports the use of these modalities in elderly patients. HT provided better UI and OAR sparing than IMRT. The T category and the RT completion time (longer than 8 weeks) were statistically significantly associated with OS.

The RTOG 97-06 study showed that RT given concurrently with or without chemotherapy provided benefits for locally advanced bladder cancer patients [29]. For elderly patients with locally advanced bladder cancer, several reports concluded that RT also was an effective treatment option for elderly patients who were not suitable for cystectomy. Santacaterina et al. [30] reported that elderly patients with muscle-invasive bladder cancer who underwent RT had a median survival of 21.5 months. Additionally, Sengelov and coworkers [31] confirmed that curative intended radiotherapy is feasible in elderly patients, with 29% surviving for 2 years. The overall actuarial median survival under 2DRT techniques in these reports ranged from 9 to 21.5 months [30,32,33]. In our institute, the median survival is 21 months. The actuarial 2-year OS, DFS, LRPFS, and MFS rates in the study were 33%, 64%, 85%, and 59%, respectively. (Figure 1, Figure 2, Figure 3, Figure 4) These dates are compatible with the previous reports suggesting IMRT and HT are feasible for elderly patients with locally advanced bladder cancer.

RT concurrent with chemotherapy, or alone, provides benefits for locally advanced bladder cancer patients. However, patients developed grade 3 or 4 hematologic toxicity or pelvic toxicities in the studies where radiation was delivered by conventional RT techniques. In the RTOG 95-06 study, 21% of patients with muscle-invading bladder cancer who underwent TURBT plus CCRT had grade 3 or 4 hematologic toxicity and 15% had grade 3 bowel toxicity [11]. Among the bladder cancer patients treated with RT or CCRT after TURBT, 25% had grade 3/4 hematologic toxicities and 10% had grade 3/4 bowl toxicities [18]. Similar results were also reported by Hagan et al [29]. In induction and consolidation regimens, the percentages of grade 3/4 hematologic toxicities were 11%/2% and 11%/0%, respectively. The grade 3/4 bowel toxicity rate in induction and consolidation regimens was 9%/0% and 0%/4%, respectively.

In the current study, none of elderly patients suffered from grade 3 or 4 acute bowel toxicities. IMRT and HT had statistically significantly better organ sparing results than 2DRT (Table 2). van Rooijen DC et al. [34] also mentioned the similar report with IMRT for bladder cancer that a statistically significant dose decrease to the small intestines can be achieved while covering both tumour and elective PTV adequately. In addition, HT had better OAR sparing ability than IMRT did in the current study. (Figure 5) Three of 19 patients (16%) experienced grade 3/4 anemia, two in the IMRT group and one in the HT group. Two of 19 patients (11%) experienced grade 3 leukopenia in the IMRT group. We believe that IMRT or HT has potential benefits for reducing the toxicities caused by 2DRT.

Dose homogeneity is a part of objective function and IMRT plan optimization is aimed at improving the value of the objective function. Dose CI is not included as a part of the objective function. The CI is usually larger than 1, indicating that a portion of the prescription dose was delivered outside the PTV. The greater CI is the less dose conformity to the PTV and a greater UI indicates higher heterogeneity in the PTV [27,28]. Comparing 2DRT, IMRT, and HT for UI and CI, both IMRT and HT showed the better conformality than 2DRT (*p *< 0.001). HT provided the better homogeneity than IMRT (*p *= 0.001) and 2DRT (*p *= 0.002). Among the patients, the additional freedom in inverse planning optimization of 51 beam angles for HT usually results in a more uniform target dose, and better avoidance of OARs compared to IMRT (Table 2).

Several studies show that the most important factor affecting treatment outcome in bladder cancer is T-stage [18,35-37]. Rodel et al. [18] noted that overall survival at 5 and 10 years was 75% and 51% for T1 tumors and 45% and 29% for muscle invasive disease, respectively. Cowan et al. [35] found that the 5-year OS rates for patients with bladder cancer were 70% for T2 disease and 51% for T3 disease. Shipley [36] also noted that the 5-year actuarial overall survival rates for T2 and T3-T4a were 62% and 41%, respectively. The 5-year overall survival rates for T1-T3a and T3b-T4b disease reported by Fokdal et al. [37] were 31% and 3%, respectively. In the current study, the 2-year OS rates for T1/2 *vs*. T3/4 disease were 66.7% *vs*. 35.4% (*p *= 0.046). We also confirmed that survival rates of elderly bladder cancer patients are related to T-stage.

RT treatment duration is a prognostic factor for OS of head and neck cancer. Langendijk et al. [38] reported that the OS rate with RT treatment durations ≤ 8 weeks and > 8 weeks were 52% and 16%, respectively. We also saw a similar phenomenon in our study of elderly bladder cancer patients. When the RT completion time is > 8 weeks patients have poorer 2-year OS rates than when RT treatment time is ≤ 8 weeks (0% vs. 37.9%, *p *= 0.004).

## Conclusions

Among our 19 patients, IMRT and HT dosimetry and organ sparing capability were superior to that of 2DRT. Additionally, IMRT and HT both produce minimal grade 3 or greater toxicity and provide good LRPFS. The T category and the RT completion time would affect the OS of bladder cancer. Long-term follow-up is needed to confirm these preliminary findings.

## Competing interests

We have no personal or financial conflict of interest and have not entered into any agreement that could interfere with our access to the data on the research, or upon our ability to analyze the data independently, to prepare manuscripts, and to publish them.

## Authors' contributions

All authors read and approved the final manuscript. CHH and PWS carried out all CT evaluations, study design, target delineations and interpretation of the study. CHH drafted the manuscript. SDC, PHC, SKL, HCC, CHH and LJW took care of patients. NSC carried out RT planning and data collection. YJC participated in manuscript preparation. LYW and YPH gave advice on the work and carried out statistical analysis.
